# Tailoring the Nonlinear Optical Response of Some Graphene Derivatives by Ultraviolet (UV) Irradiation

**DOI:** 10.3390/nano12010152

**Published:** 2022-01-01

**Authors:** Aristeidis Stathis, Zoi Bouza, Ioannis Papadakis, Stelios Couris

**Affiliations:** 1 Department of Physics, University of Patras, 26504 Patras, Greece; a.stathis@iceht.forth.gr (A.S.); z.bouza@upnet.gr (Z.B.); j.papadakis@iceht.forth.gr (I.P.); 2 Institute of Chemical Engineering Sciences (ICE-HT), Foundation for Research and Technology-Hellas (FORTH), 26504 Patras, Greece

**Keywords:** UV irradiation, graphene, fluorographene, graphene oxide, hydrogenated fluorographene, nonlinear optical response, graphene functionalization

## Abstract

In the present work the impact of in situ photoreduction, by means of ultraviolet (UV) irradiation, on the nonlinear optical response (NLO) of some graphene oxide (GO), fluorographene (GF), hydrogenated fluorographene (GFH) and graphene (G) dispersions is studied. In situ UV photoreduction allowed for the extended modification of the degree of functionalization (i.e., oxidization, fluorination and hydrogenation), leading to the effective tuning of the corresponding sp^2^/sp^3^ hybridization ratios. The nonlinear optical properties of the studied samples prior to and after UV irradiation were determined by means of the Z-scan technique using visible (532 nm), 4 ns laser excitation, and were found to change significantly. More specifically, while GO’s nonlinear optical response increases with irradiation time, GF and GFH present a monotonic decrease. The graphene dispersions’ nonlinear optical response remains unaffected after prolonged UV irradiation for more than an hour. The present findings demonstrate that UV photoreduction can be an effective and simple strategy for tuning the nonlinear optical response of these graphene derivatives in a controllable way, resulting in derivatives with custom-made responses, thus more suitable for different photonic and optoelectronic applications.

## 1. Introduction

During the last decade graphene [1] has been among the most studied materials due to its extraordinary properties. Graphene is a two-dimensional carbon allotrope with zero bandgap [2], while its unique mechanical, chemical, and opto-electronic properties render it a highly attractive material for a plethora of emerging applications, related with solar cells [3], photonic sensors [4], supercapacitors [5,6], flexible electronics [7,8], field-effect transistors [9] and several others.

However, the zero bandgap of graphene hinders its integration into specific electronic and optoelectronic devices [4]. In that view, significant research efforts have been oriented towards possible ways to open and control a finite energy gap in the band structure of graphene, enlarging its potential for more applications. So far, various methods have been proposed in the literature, such as applying strain along the graphene sheet [10,11], biasing bilayers of G [12,13], substrate-induced bandgap supported on SiC [14], patterned hydrogen adsorption in the graphene sheet [15] and so forth. Among the different strategies proposed for the modification of graphene’s energy bandgap, maybe the most often used one is its covalent functionalization through the introduction of suitable atoms on the basal plane of the graphene sheet [16,17]. Covalent functionalization takes advantage of the fact that graphene’s carbon atoms are sp^2^-hybridized and each carbon has a p_z_ orbital in the direction perpendicular to the basal plane, forming a relative stable conjugated π-bond [17]. Recently, various atoms and organic/inorganic molecules have been proposed and extensively used for the functionalization of graphene [18,19,20]. Overall, these studies have confirmed that the modification of graphene’s surface with various atoms/molecules could alter the properties of graphene substantially, enabling the creation of a wide portfolio of newly synthesized graphene derivatives with tunable optical, chemical and mechanical properties. Two of the most studied graphene derivatives are graphene oxide [21] and fluorographene [22,23].

Graphene oxide (GO) is a key functionalized analogue of graphene, obtained by exfoliating graphite with strong oxidizing agents [21]. In contrast to G, where the strong interlayer van der Waals forces do not allow the formation of stable dispersions in common organic solvents, GO is considered among the most used materials because of its very good dispersibility in solvents [24], which provides much easier handling and applicability, while the presence of hydroxyl, epoxy, phenolic and carboxyl functionalities enriches its chemistry for further derivation [25]. GO exhibits a bandgap that can be varied over a wide range, e.g., from about 0.5 to 2.2 eV [26], by modifying the amount and type of oxygen-containing groups [27,28], making GO behave either as a semiconductor or a semi-metal [29].

On the other hand, graphene fluoride, or fluorographene (GF), is a two-dimensional wide-bandgap semiconductor (3 eV) which is optically transparent in the visible part of the optical spectrum [30]. The chemical modification of graphene fluoride takes place easily via controllable substitution/defluorination [22,23], leading to reconstructive derivatives with a desired fluorine content [31] and providing tunability of the optical and chemical properties, rendering graphene fluoride as one of the most promising precursors for novel graphene derivatives.

A new member of the fluorographene derivatives is hydrogenated fluorographene (GFH), which can be considered as the 2D counterpart of graphane (hydrogenated graphene) [32]. It is directly derived from GF through a hydride reaction and contains both sp^3^ and sp^2^ carbon domains. The sp^3^ carbon domains are related to the newly inserted C-H groups and residual C-F groups after hydride substitution, whereas the sp^2^ carbon domains are related to graphenic domains formed by the partial hydride reduction of GF. In the same way, as in the case of GF, the ability to easily tune the hybridization by simply changing the F and H contents of GFH can greatly affect its bandgap and therefore its NLO response.

From the above, it can be easily understood that an efficient and relatively simple way to tailor the energy bandgap of these chemically modified graphenes (i.e., GO, GF and GFH) is by modifying their oxygen/fluorine content, i.e., the sp^2^/sp^3^ hybridization ratio. The ability to tune this ratio allows for the continuous engineering of the bandgap.

With that view, in the present work the 355 nm third harmonic output of a 4 ns Q-switched Nd:YAG laser was employed to irradiate some GO, GF and GFH dispersions for different irradiation periods and laser fluences, aiming to photo-induce significant changes in the sp^2^/sp^3^ ratio of these graphene derivatives and modify their third-order nonlinear optical (NLO) response. The NLO responses of the dispersions were then studied in detail by means of the Z-scan technique using the second harmonic output at 532 nm of the same laser. For completeness, pristine graphene (G) dispersions were also prepared and irradiated by the 355 nm UV laser light and their NLO responses were evaluated under the same experimental conditions used for the derivatives.

## 2. Materials and Methods

### 2.1. Synthesis and Characterization

Graphene (G) was obtained by liquid-phase exfoliation of graphite in dimethylformamide (DMF) [33,34]. The so-prepared G samples consisted of 2–5 layers [35]. GO was obtained through the oxidation of natural graphite powder (Sigma-Aldrich, St. Louis, MO, USA) using the method described by Marcano et al. [21], resulting in an O/C ratio of 0.25. The oxidation degree of the samples was confirmed by means of high-resolution X-ray photoelectron spectroscopy (HR-XPS), allowing for the determination of the oxygen and sp^2^ carbon content [36]. Fluorographene (GF) was obtained through the sonication of fluorinated graphite (Sigma Aldrich, St. Louis, MO, USA), resulting in fully fluorinated graphene with a nominal atomic composition of F/C = 1:1 as confirmed by XPS elemental analysis, whereas atomic force microscopy (AFM) measurements revealed a layer thickness of 2–2.5 nm [32]. GFH was obtained by a two-step synthetic procedure. Firstly, liquid-phase exfoliation of fluorinated graphite (C/F = 1:1 ratio, Sigma Aldrich, St. Louis, MO, USA) in DMF was applied, which resulted, after sonication, in GF sheets. Lastly, the GF sheets were hydrogenated by means of hydride nucleophilic substitution/reduction of the GF layers using sodium borohydride (NaBH_4_) as the hydride source [32]. XPS measurements revealed a stoichiometry of C_18_H_2.2_F_2.8_O_1.3_, leading to an estimation of graphene’s total functionalization of ~35%, with ~12% and ~15% of H atom and F atom coverages, respectively. AFM measurements showed a thinner layer thickness of 0.8–1 nm compared to its precursor GF. More details on the synthesis along with various characterization techniques of the investigated samples can be found in detail elsewhere [32,34,36].

### 2.2. In Situ UV Irradiation of G, GO, GF and GFH

For the photo-reduction experiments, dispersions of G and GF in DMF were prepared while GO was dispersed in distilled water and GFH in acetone. All prepared dispersions were routinely checked spectrophotometrically before the measurements and were found to be stable. All solvents used were spectrophotometric-grade. For all the experiments, the dispersions were placed in 1-mm-thick glass cells and irradiated by the third harmonic output at 355 nm of a 4 ns Q-switched Nd:YAG laser. The laser beam was slightly expanded by means of a homemade telescope to obtain a homogeneous illumination of the frontal surface of the cell containing the sample. The energies of the incident and the transmitted laser beam were measured by a calibrated joulemeter (Coherent-EnergyMax J-10MT-10kHZ, Coherent Inc., Santa Clara, CA, USA) placed just before and behind the cell, respectively. Each sample was exposed to different duration irradiation periods (i.e., cycles), lasting from a few minutes up to one hour. In the following sections the irradiated samples will be indicated as iGX-y, where X denotes O, F or FH and y denotes the duration of irradiation. The UV–VIS–NIR absorption spectra of the prepared dispersions were measured systematically by means of a double-beam spectrophotometer (V-670 Jasco, JASCO, Easton, MD, USA) in the spectral region from 200 to 1200 nm, before and after each irradiation cycle. The UV irradiation of the dispersions was repeated until no significant changes were observed in their UV–VIS–NIR absorption spectra.

### 2.3. Nonlinear Optical Measurements

**Figure 1:**
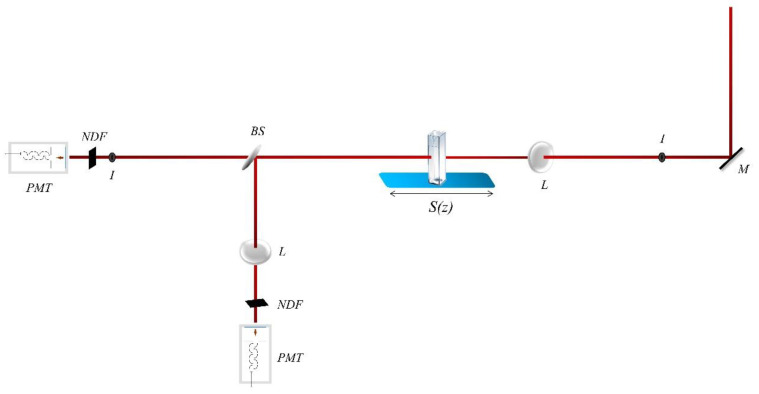
Configuration of the Z-scan technique.

The third-order nonlinear optical (NLO) response of G, GO, GF and GFH has been investigated using the Z-scan technique [37], which allows the simultaneous determination of the sign and the magnitude of the nonlinear absorption (i.e., nonlinear absorption coefficient β) and refraction (i.e., nonlinear refractive index parameter γ′) of a sample from a single transmission measurement. In more detail, in the Z-scan technique the transmission of a sample moving along the propagation direction of a focused laser beam, thus experiencing a different laser intensity at each position, is measured. The experimental setup is schematically depicted in Figure 1.

As the sample approaches the focal plane it experiences continuously increasing laser intensity, which gives rise to nonlinear absorption and refraction, which in turn modify its transmission characteristics. The assessment of the nonlinear absorption and refraction of the sample is achieved by splitting the transmittedthrough the sample laser beam in two parts and introducing each part into the so-called “open-aperture” (OA) and “closed-aperture” (CA) Z-scan arms, respectively.

In the OA Z-scan arm the transmitted laser light is totally collected and measured, e.g., by means of a photomultiplier (PMT). According to Z-scan formalism the sample’s normalized transmission T(z) is described by the following equation:

(1) $$Τ(z)=\frac{1}{\sqrt{\pi }\left[\frac{{\beta Ι}_{0}L_{\mathrm{eff}}}{\left({1+z}^{2}/z_{0}^{2}\right)}\right]}\int_{-\infty }^{+\infty }\ln\left[1+\frac{{\beta Ι}_{0}L_{\mathrm{eff}}}{\left({1+z}^{2}/z_{0}^{2}\right)}e^{-t^{2}}\right]\mathrm{dt}$$

where β is the nonlinear absorption coefficient, I_0_ is the on-axis peak irradiance, z_0_ is the Rayleigh length (or confocal parameter) and $L_{\mathrm{eff}}=\left[1-e^{-\alpha_{0}L}\right]/\alpha_{0}$, with α_0_ being the linear absorption coefficient at the laser wavelength and L denoting the thickness of the sample.

From the fitting of the OA Z-scan data with Equation (1), the nonlinear absorption coefficient β can be obtained. The presence of a minimum or maximum at the OA Z-scan indicates the sign of the nonlinear absorption coefficient β, corresponding to reverse saturable absorption (RSA, β > 0) or saturable absorption (SA, β < 0), respectively. Then, the imaginary part of the third-order nonlinear susceptibility χ^(3)^, Imχ^(3)^, can be calculated from the following relation:

(2) Imχ(3)(esu)=10−7c2n0296π2ωβ(cm/W)

where c is the speed of light given in cm s^−1^, n_0_ is the refractive index and ω is the frequency of the incident beam in s^−1^.

In the CA Z-scan arm the transmitted through the sample laser beam is passed firstly through a narrow pinhole placed in the farfield and is subsequently measured, e.g., by a second PMT. This type of transmission measurement provides information about the nonlinear refractive index parameter γ′. A CA Z-scan can exhibit a pre-focal transmission minimum (valley) followed by a post-focal maximum (peak) or the opposite configuration (i.e., a peak followed by a valley), indicating positive or negative γ′ (i.e., Reχ^(3)^), respectively. In the former case the sample acts as a focusing (positive γ′) lens while in the latter case the sample acts as a defocusing (negative γ′) lens.

The nonlinear refractive index parameter γ′ can be determined by fitting the corresponding CA Z-scan curve using the following equation:

(3) T(z)=1+4γ′k0Leffzz0(1+z2/z02)(9+z2/z02)

where $k_{0}=\frac{2\pi }{\lambda }$ is the wavevector and λ is the laser wavelength, all in free space. The quantities z_0_ and L_eff_ are defined as previously.

From the determined nonlinear refractive index parameter γ′, the real part of the third-order nonlinear susceptibility χ^(3)^, Reχ^(3)^, can be calculated from the following relation:

(4) Reχ(3)(esu)=10−6cn02480π2γ′(cm2/W)

Finally, the magnitude of the third-order nonlinear susceptibility χ^(3)^ can be easily calculated as follows:

(5) χ(3)=(Reχ(3))2+(Imχ(3))2

For the nonlinear optical measurements, the second harmonic output at 532 nm from a 4 ns Q-switched Nd:YAG laser was used. The laser was operating at a repetition rate of 1 to 10 Hz. The laser beam was focused into the sample by means of a 20 cm focal length quartz lens. The laser beam waist at the focus (i.e., half width at 1/e^2^ of the maximum of irradiance) was determined using a CCD camera. It was found to be 17.5 μm.

## 3. Results and Discussion

### 3.1. UV Irradiation Experiments of the G, GF, GO and GFH Dispersions

**Figure 2:**
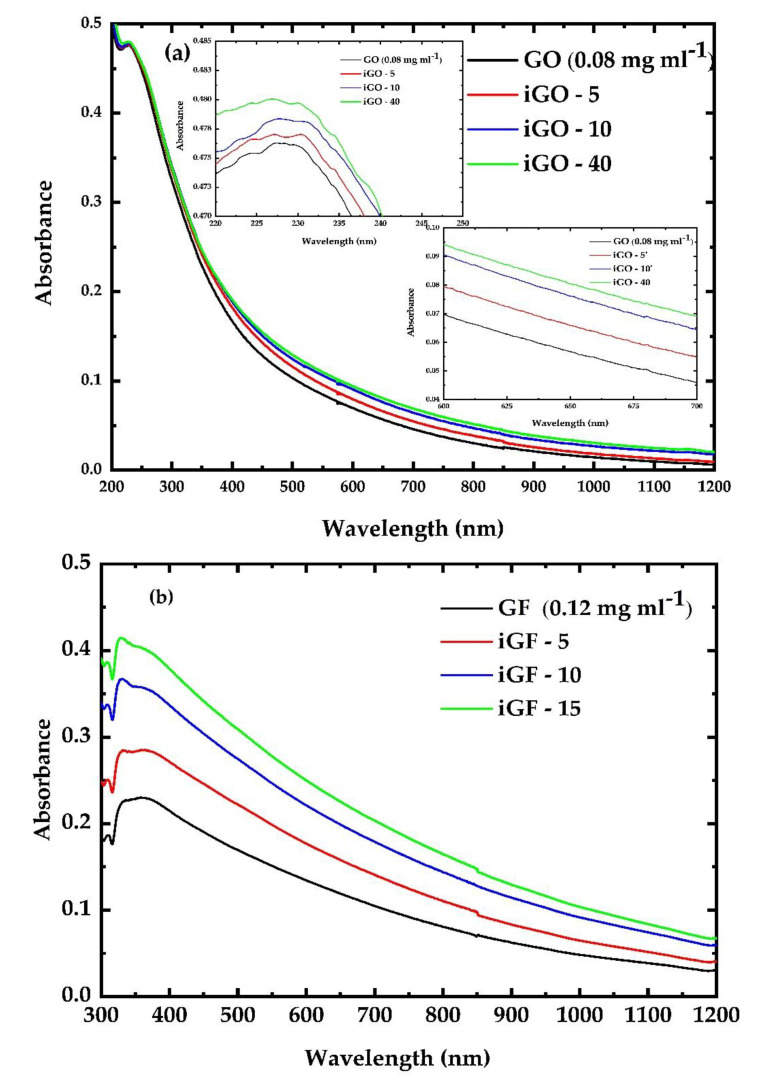

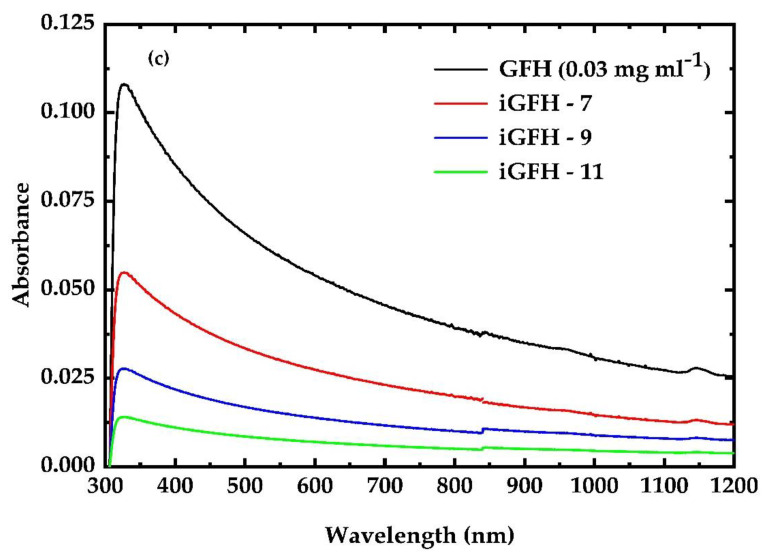
Evolution of the UV–Vis–NIR absorption spectra of non-irradiated and irradiated (**a**) GO, (**b**) GF and (**c**) GFH dispersions under UV irradiation.

The effects of the ultraviolet irradiation on the G, GO, GF and GFH dispersions were monitored through the collection and study of their respective UV–VIS–NIR absorption spectra after each irradiation cycle. By monitoring the spectral changes, evidence about the induced structural changes that resulted from the UV irradiation can be obtained. Figure 2 presents the UV–VIS–NIR absorption spectra of some GO (Figure 2a), GF (Figure 2b) and GFH (Figure 2c) dispersions prior to and after three successive irradiation cycles. As shown, the absorption of GO and GF dispersions was found to increase with the duration of UV irradiation, while the absorption of the GFH dispersion was found to decrease with the duration of its exposure to the UV light. It should be mentioned here that the laser fluences used for the irradiation experiments were chosen based on how strong the induced changes were in the respective absorption spectra. Therefore, when large absorption changes were observed the laser fluence was reduced accordingly to better monitor the temporal evolution of the induced absorption changes. To ensure the validity of the experimental observations, for each sample four identical dispersions were prepared and irradiated consecutively under identical conditions. This similarly occurred for the studies of the NLO response which will be presented in the next.

More specifically, the GO dispersions were initially irradiated for two sequential cycles of 5 min each. After each irradiation cycle a small but clearly observable absorption change (increase) was observed, as shown in the bottom inset of Figure 2a. Then, the GO sample was further irradiated for a 30 min cycle and a relatively larger absorption change was observed. Further UV irradiation of the GO dispersion, i.e., beyond the three irradiation cycles (having lasted 40 min totally), did not result in an appreciable change in its absorption. For all irradiation cycles, the laser fluence was 13 mJ cm^−2^.

As can be seen from Figure 2a, the absorption spectra of the GO dispersions are structureless, presenting a characteristic absorption band at ~227 nm ascribed to π–π* transitions [38]. Upon irradiation this characteristic absorption was observed to shift gradually to longer wavelengths (red-shifted), suggesting a reduced energy of the π–π* transitions, associated with the increased conjugation of the irradiated iGOy graphenic sheets [38]. An analogous behavior has been reported by Trusovas et al. [39]; it has been attributed to the reduction of GO to graphene induced by ps laser irradiation, resulting in the increased conjugation of iGOy sheets and suggesting the creation of more conductive sp^2^ graphenic domains on the insulating GO [40]. Similar observations have been reported by Wu. et al. [41], having used a 200 W mercury lamp to prepare reduced GO (RGO) samples. It should be noted at this point that since the present GO samples were partially oxidized graphene, with an oxygen/carbon atomic ratio (O/C) of ~0.25, the effects of the UV irradiation on the absorption spectrum were not so pronounced, at least not after the first two irradiation cycles. In any case, an increase in the exposure time resulted in a decrease in the degree of oxidation, leading the GO dispersions to exhibit a more “graphenic” behavior. This is further supported by the bandgap values of the irradiated GO samples, as estimated using the Tauc method. Although the accuracy of this method can be limited, it provides an easy way for such an estimation by expressing the linear absorption coefficient, α_0,_ through the following equation:

(6) (a0·hv)1n=(hv−Eg)

where *h* is the Planck constant, *v* is the photon’s frequency and *E_g_* is the bandgap energy, while the parameter n depends on the nature of the electron transition and takes the value of ½ for the case of direct bandgaps. Therefore, the bandgap value of the non-irradiated GO was found to be ~3.76 eV, which is in relatively good agreement with other values from the literature [26]. In general, the UV photo-reduction led to a monotonic decrease in the bandgap, from ~3.70 to ~3.61 for iGO-5 and iGO-40 dispersions, respectively, providing evidence that the degree of oxidation is decreases upon irradiation, the sample exhibiting a more “graphene”-like behavior.

Concerning GF, the corresponding dispersions were subjected to three successive UV irradiation cycles of 5 min each, i.e., corresponding to a total exposure time of 15 min. The laser fluence was set at 5 mJ cm^−2^. As can be seen from Figure 2b, the UV–VIS–NIR absorption spectrum of GF was structureless, exhibiting a strong absorption band at ~330 nm which is attributed to the π–π* transitions of C=C. The UV irradiation of the GF dispersions led to a monotonic increase in the 330 nm absorption band, indicating the de-fluorination of GF and thus the change of the conjugation on the GF sheet, the latter gradually recovering, partially, to a “graphenic” sheet [24,42]. Therefore, as the duration of the UV irradiation increased the degree of fluorination decreased, corresponding to an increase in the sp^2^-hybridized graphenic domains (C-C) against the sp^3^ domains (C-F), finally resulting in an enhancement of the 330 nm absorption band. Again, the use of the Tauc method supported the monotonic decreasing trend of the bandgap values of the irradiated GF samples. Non-irradiated GF exhibited a bandgap of ~2.35 eV, similar to what has been reported in the literature [30]. As the irradiation time increased the bandgap attained a minimum value of ~2.09 eV for iGF-15, suggesting the partial recovery of the graphenic sheet. The most noticeable change in the UV–VIS–NIR absorption spectrum was observed within the first cycle (i.e., 5 min) of irradiation. This can be understood by considering the different natures of C-F bonds which are present in GF, such as the covalent and “semi-covalent” (or “semi-ionic”) ones [43]. According to the XPS findings of the deconvoluted C 1s spectrum of the studied GF [32], a minor fraction of C-F is attributed to “semi-ionic” (at 287.7 eV) bonds whereas the covalent C-F bonds (at 290.1 eV and 291.8 eV) prevail. According to Ren et al. [44] the covalent C-F bonds are more sensitive to UV radiation than semi-ionic ones. This can qualitatively explain the observation of the initially faster reduction (i.e., within the first 5 min of irradiation). After three irradiation cycles (i.e., 5 min each, 15 min in total) no significant changes were observed in the UV–VIS–NIR spectra of GF, suggesting that further de-fluorination is most probably less efficient. It should be added here that Ren et al. performed the UV irradiation of the GF samples using the 365 nm line from a mercury (Hg) discharge lamp, employing a fluence of 2.66 W cm^−2^, while in the present study radiation at 355 nm and a fluence of 5 mJ cm^−2^ were used. The present results are in very good agreement with the results of Ren et al.

Then, the GFH dispersions were UV-irradiated for three 5 min irradiation cycles using a fluence of 5 mJ cm^−2^. As shown in Figure 2c, the GFH dispersion absorption spectrum is dominated by a strong absorption band located at ~330 nm, while it is structureless towards longer wavelengths. Interestingly, in contrast to the behavior observed for the corresponding characteristic GF absorption band, an increase in the UV exposure time of the GFH dispersion resulted in a significant decrease in the GFH absorption band, initially leading to its gradual attenuation and its total extinction for irradiation durations greater than 10 min, suggesting a drastic change in the conjugation. This observation suggests the lowering of the energy bandgap. More specifically, while the energy bandgap of non-irradiated GFH is estimated to be ~2.7 eV the bandgap of the iGFH-11 was estimated to be ~2.5 eV. Moreover, it has been shown that the degree of fluorination and the conformations of the F atoms on the graphenic sheet can greatly influence the bandgap of GFH [45]. Therefore, as the irradiation time increases the degree of fluorination decreases, leading to the lowering of the bandgap value.

**Figure 3:**
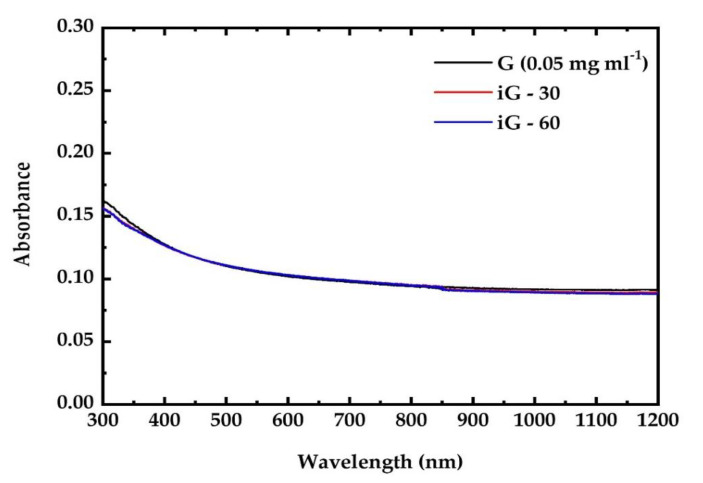
UV–Vis–NIR absorption spectra of pristine and irradiated G dispersion.

Finally, pristine graphene (G) dispersions in DMF were irradiated for various irradiation cycles with a fluence of 25 mJ cm^−2^. The corresponding UV–VIS–NIR absorption spectra are presented in Figure 3. Although graphene dispersions were irradiated for more than one hour totally, at laser fluences higher than those used for GO, GF and GFH no measurable modifications/changes were observed in their UV–VIS–NIR absorption spectra. This finding most probably indicates that no major conjugation changes in the graphenic sheet have taken place under UV irradiation.

### 3.2. Measurements of the Nonlinear Optical (NLO) Properties

**Figure 4:**
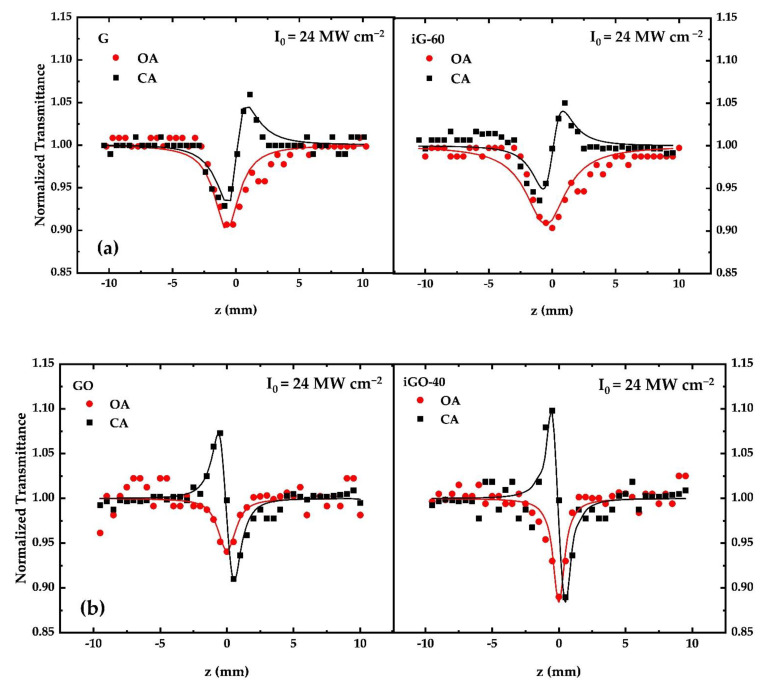

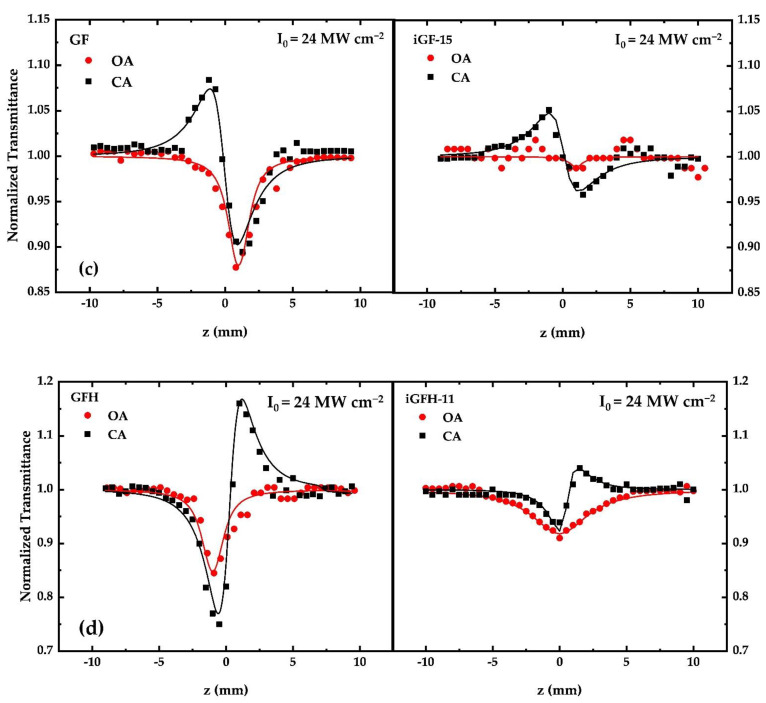
Representative Z-scan recordings showing the evolution of the NLO properties of non-irradiated and irradiated (**a**) G, (**b**) GO, (**c**) GF and (**d**) GFH dispersions.

The NLO response of each dispersion was evaluated prior to its irradiation and after each UV irradiation cycle. For the accurate determination of the NLO parameters (i.e., nonlinear absorption coefficient β, nonlinear refractive index parameter γ′) Z-scan measurements of each dispersion were performed using different incident laser intensities. It is useful to add at this point that the linear transmittance of the samples was measured prior to and after irradiation at the laser excitation wavelength. The values of the linear transmittance of the non-irradiated and irradiated G samples were determined to remain unchanged, i.e., ~77%, while those of the GO and iGO-15 samples were found to be ~81 and ~76.5%, respectively. In the case of the GF and iGF-15 samples the linear transmittance was found to be ~70 and 51% prior to and after irradiation, whereas for the GFH and iGFH-11 samples it was found to be ~98 and ~87%, respectively. As an example, some representative OA and CA Z-scans of each sample prior to and after UV irradiation are presented in Figure 4. All Z-scan curves presented in Figure 4 have been obtained using the same laser peak intensity, I_0_, i.e., I_0_ = 24 MW cm^−2^. The filled circle/square dots correspond to the experimental data points whereas the lines correspond to the theoretical fitting of the OA and CA curves by Equations (1) and (3), respectively.

It is important to note that since the solvents used for the dispersions (i.e., DMF, distilled water and acetone) did not exhibit any NLO response under the present experimental conditions the Z-scan recordings directly reveal the sign and magnitude of the NLO response of the non-irradiated and irradiated dispersions. Therefore, as shown from the Z-scan measurements, G, GO, GF and GFH were all found to exhibit an important NLO response under ns laser excitation. More specifically, all samples were found to exhibit reverse saturable absorption (RSA) behavior (i.e., Imχ^(3)^ > 0), attributed to two-photon absorption (2PA) and/or excited-state absorption (ESA), as has been discussed in detail elsewhere [46]. It is interesting to state at this point that, although saturable absorption (SA) has to be expected (due to the flat absorption spectra of all the samples in the visible region, suggesting resonant character excitation), RSA behavior was observed (for both non-irradiated and irradiated samples). This last observation suggests that the operational mechanism responsible for the NLO absorption under ns excitation conditions is most probably due to 2PA and/or ESA. This can be further understood by considering that the studied graphene samples consist of 2–5 layers (as has been described above), while layer stacking in graphene is known to induce the opening of the bandgap [13]. Thus, the observed RSA behavior can be explained in terms of a 2PA mechanism. Therefore, although 2PA is absent in monolayer graphene (since it possesses a near-zero bandgap), a 2PA mechanism can occur in bilayer and multilayer graphene due to new states introduced by the π–π stacking of the layers, thus explaining the RSA behavior. In support of the above, it is useful to add that in another investigation (unpublished results), concerning the NLO response under 35 fs, 800 nm laser excitation of some CVD-grown monolayer, bilayer and trilayer graphene films, similar findings have been observed, i.e., the monolayer graphene samples exhibited SA behavior for a very wide range of laser intensities, while the bilayer and trilayer samples initially exhibited SA behavior, which turned into RSA behavior (attributed to 2PA) at a higher laser intensity.

Concerning the NLO refractive response of the studied dispersions, the CA Z-scans of the GO and GF dispersions exhibited a peak–valley configuration, indicating self-defocusing behavior (i.e., Reχ^(3)^ < 0), while G and GFH exhibited a valley–peak configuration, indicating self-focusing behavior (i.e., Reχ^(3)^ > 0). The sign of the CA Z-scan recording remained unaltered before and after irradiation.

From the obtained Z-scans the values of the NLO parameters were determined; they are summarized in Table 1. To facilitate comparisons the values of the different NLO parameters listed in this table are all referring to a concentration of 1 mg mL^−1^. As can be seen from this table, the NLO response of GO was found to increase upon irradiation, exhibiting a three-fold increase. The observed increase in the NLO response is associated with the extension of the π-conjugation along the graphenic sheet, as has been discussed in detail by Liaros et al. [47] who concluded that GO samples with a lower degree of oxidation (either prepared with a lower degree of oxidation or UV-reduced GO samples) exhibited a larger NLO response due to possessing more sp^2^ sites.

In contrast to the NLO response of GO, GF’s NLO response was found to decrease upon irradiation. Specifically, GF’s NLO response was monotonically decreased, attaining almost a two-fold decrease at the end of its total irradiation. This decrease can be understood by considering GF’s stoichiometry. The studied GF has a C/F ratio of 1:1, i.e., every carbon atom is bonded to one F atom (or with two or three F atoms at the edges of the sheet). This suggests that there are not any sp^2^ carbons in the non-irradiated sample. UV irradiation induces reductive defluorination, leading to the creation of sp^2^ domains within the sp^3^ network. Therefore, as UV irradiation proceeds GF’s sp^2^/sp^3^ ratio increases, modifying the NLO properties [46].

The most remarkable variation in the NLO response upon UV irradiation was observed for the case of GFH dispersions, where the NLO response was found to be reduced to half after each 2 min irradiation cycle, eventually attaining an eight-fold decrease (i.e., after 11 min of total irradiation). This finding can be attributed to the increase in the sp^2^/sp^3^ hybridization ratio of the irradiated dispersions [46]. More specifically, as the irradiation time increases the conjugation of the GFH samples changes, mainly because of the occurring de-fluorination. Therefore, domains that were (initially) dominated by sp^3^ hybridization are transformed into domains with sp^2^ hybridization. As a result, the ratio of sp^2^/sp^3^ increases and the irradiated samples acquire a more “graphenic” behavior, accompanied by a reduction in their energy bandgap (from ~2.7 to ~2.5 eV). Thus, the NLO response is greatly modified between non-irradiated and irradiated samples.

**Figure 5:**
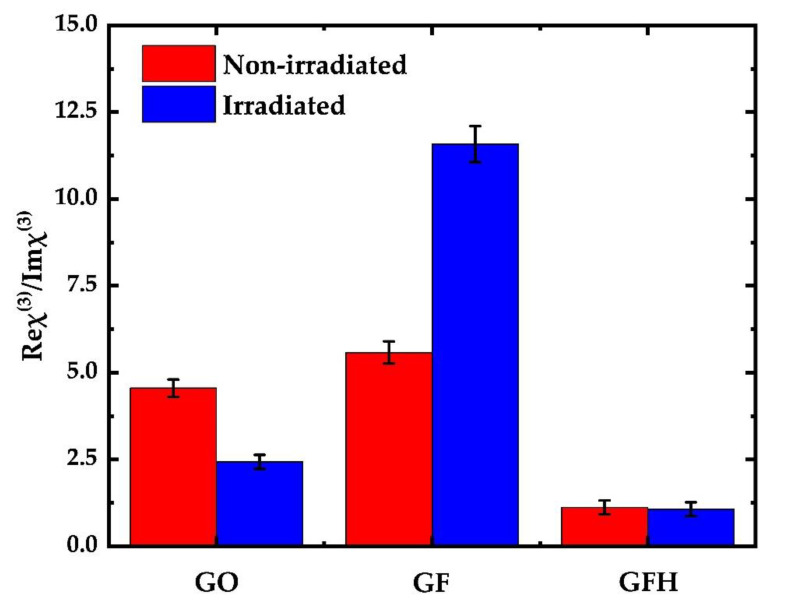
Variation in the Reχ^(3)^/Imχ^(3)^ ratio for each sample before and after irradiation.

Another interesting observation can be made by comparing the ratio of the NLO absorption and refraction (Reχ^(3)^/Imχ^(3)^) of each sample prior to and after irradiation. The corresponding ratio for each sample is presented in Figure 5. The red and blue bars indicate the ratio of Reχ^(3)^/Imχ^(3)^ prior to and after irradiation. In the cases of GO and GF dispersions the Reχ^(3)^/Imχ^(3)^ ratio was about 5, whereas it was ~1 for GFH. After the completion of UV irradiation this ratio was found to increase, becoming greater than 10 for the case of GF. Oppositely, in the case of GO this ratio decreased to a value of ~2. Interestingly, this ratio remains the same for GFH. In conclusion, the present findings indicate that the NLO absorption of GO and GF were more affected by UV irradiation than the corresponding NLO refraction. In the case of GFH UV irradiation affected similarly both the NLO absorption and refraction. The modification of the relative strength between the NLO refraction and absorption by means of in situ UV photo-reduction can be a further auxiliary and efficient way to construct graphene derivatives with custom-made NLO properties in view of specific optoelectronic applications.

Finally, the NLO response of graphene dispersions was found to remain unaltered even after 60 min of UV irradiation, a finding which is fully compatible with the measured UV–VIS–NIR spectra of graphene’s dispersions, which remained unchanged upon irradiation.

## 4. Conclusions

In summary, in the present work the UV photoreduction of some graphene (G), graphene oxide (GO), graphene fluoride (GF) and hydrogenated graphene fluoride (GFH) dispersions is investigated for the engineering of the third-order NLO response of these graphene derivatives. The UV-induced photoreduction was achieved by using the third harmonic output, at 355 nm, from a 4 ns Nd:YAG laser. Then, the third-order NLO response of the samples was investigated using the Z-scan technique employing the second harmonic output, at 532 nm, from the same nanosecond Nd:YAG laser. The comparison of the UV–VIS–NIR absorption spectra of several dispersions of G, GO, GF and GFH samples prior to and after UV irradiation showed that GO, GF and GFH dispersions exhibited significant changes, reflecting the resulting changes in the conjugation of the graphenic sheet, while the dispersions of pristine graphene remained unaltered. The NLO response of the irradiated, i.e., photo-reduced, samples were found to vary significantly upon irradiation time, reflecting the structural changes occurring. In particular, the NLO response of GO dispersions was found to increase upon irradiation, in contrast to the monotonically decreasing NLO response of GF and GFH dispersions, while the NLO response of pristine graphene dispersions remained unaffected. The present experimental findings clearly suggest that UV photoreduction can be a useful and efficient strategy for the controlled tailoring of the nonlinear optical properties of these graphene derivatives, leading to a wide portfolio of graphene-based materials with custom-made nonlinear optical responses, thus materials that better serve the needs for several photonic and optoelectronic applications.

## Figures and Tables

**Table 1:** NLO parameters of G, GO, GF and GFH before and after UV irradiation. All values refer to a concentration of 1 mg mL^−1^.

Sample	β (×10^−11^ m W^−1^)	γ′ (×10^−18^ m^2^ W^−1^)	Imχ^(3)^ (×10^−13^ esu)	Reχ^(3)^ (×10^−13^ esu)	|χ^(3)^| (×10^−13^ esu)
G	1240 ± 149	1440 ± 173	680 ± 79	1880 ± 221	2000 ± 313
GO	44 ± 5	−81 ± 8	20 ± 2	−91 ± 9	95 ± 9
iGO-5	81 ± 10	−100 ± 9	37 ± 4	−112 ± 10	118 ± 11
iGO-10	138 ± 12	−125 ± 15	63 ± 6	−140 ± 17	154 ± 18
iGO-40	263 ± 20	−263 ± 24	121 ± 9	−294 ± 27	318 ± 28
GF	683 ± 72	−1583 ± 177	367 ± 38	−2050 ± 226	2083 ± 230
iGF-5	475 ± 60	−1317 ± 145	250 ± 32	−1708 ± 185	1725 ± 187
iGF-10	333 ± 32	−1083 ± 115	175 ± 17	−1408 ± 147	1417 ± 148
iGF-15	192 ± 18	−900 ± 85	100 ± 9	−1158 ± 108	1168 ± 109
GFH	8067 ± 811	3700 ± 375	3867 ± 390	4333 ± 439	5807 ± 598
iGFH-7	4000 ± 254	1800 ± 235	1933 ± 122	2107 ± 298	2867 ± 322
iGFH-9	2000 ± 187	900 ± 90	967 ± 90	1067 ± 105	1433 ± 138
iGFH-11	1033 ± 120	467 ± 41	497 ± 57	533 ± 48	730 ± 74

## Data Availability

Not applicable.

## References

[1] Novoselov K., Geim A., Morozov S., Jiang D., Zhang Y., Dubonos S., Grigorieva I., Firsov A. (2004). Electric Field Effect in Atomically Thin Carbon Films. Science.

[2] Castro Neto A.H., Guinea F., Peres N.M.R., Novoselov K., Geim A.K. (2009). The electronic properties of graphene. Rev. Mod. Phys..

[3] Iqbal M.Z., Rehman A.-U. (2018). Recent progress in graphene incorporated solar cell devices. Sol. Energy.

[4] Bao Q., Loh K.P. (2012). Graphene Photonics, Plasmonics, and Broadband Optoelectronic Devices. ACS Nano.

[5] Bokhari S.W., Siddique A.H., Sherrell P.C., Yue X., Karumbaiah K.M., Wei S., Ellis A.V., Wei G. (2020). Advances in graphene-based supercapacitor electrodes. Energy Rep..

[6] Yang W., Ni M., Ren X., Tian Y., Li N., Su Y., Zhang X. (2015). Graphene in Supercapacitor Applications. Curr. Opin. Colloid Interface Sci..

[7] Dai C., Sun G., Hu L., Xiao Y., Zhang Z., Qu L. (2020). Recent progress in graphene-based electrodes for flexible batteries. Infomat.

[8] Han T.-H., Kim H., Kwon S.-J., Lee T.-W. (2017). Graphene-based flexible electronic devices. Mater. Sci. Eng. R Rep..

[9] Novodchuk I., Bajcsy M., Yavuz M. (2021). Graphene-based field effect transistor biosensors for breast cancer detection: A review on biosensing strategies. Carbon.

[10] Ni Z., Yu T., Lu Y., Wang Y., Feng Y., Shen Z. (2008). Uniaxial Strain on Graphene: Raman Spectroscopy Study and Band-Gap Opening. ACS Nano.

[11] Mohiuddin T., Lombardo A., Nair R., Bonetti A., Savini G., Jalil R., Bonini N., Basko D., Galiotis C., Marzari N. (2009). Uniaxial strain in graphene by Raman spectroscopy: G peak splitting, Grüneisen parameters, and sample orientation. Phys. Rev. B.

[12] Ohta T., Bostwick A., Seyller T., Horn K., Rotenberg E. (2006). Controlling the Electronic Structure of Bilayer Graphene. Science.

[13] Zhang Y., Tang T., Girit C., Hao Z., Martin M., Zettl A., Crommie M., Shen Y., Wang F. (2009). Direct observation of a widely tunable bandgap in bilayer graphene. Nature.

[14] Zhou S., Gweon G., Fedorov A., de Heer F.P., Lee W., Guinea F., Castro Neto A.H., Lanzara A. (2007). Substrate-induced bandgap opening in epitaxial graphene. Nat. Mater..

[15] Balog R., Jørgensen B., Nilsson L., Andersen M., Rienks E., Bianchi M., Fanetti M., Lægsgaard E., Baraldi A., Lizzit S. (2010). Bandgap opening in graphene induced by patterned hydrogen adsorption. Nat. Mater..

[16] Park J., Yan M. (2012). Covalent Functionalization of Graphene with Reactive Intermediates. Acc. Chem. Res..

[17] Pašti I., Jovanović A., Dobrota A., Mentus S., Johansson B., Skorodumova N. (2018). Atomic adsorption on graphene with a single vacancy: Systematic DFT study through the periodic table of elements. Phys. Chem. Chem. Phys..

[18] Georgakilas V., Otyepka M., Bourlinos A., Chandra V., Kim N., Kemp K., Hobza P., Zboril R., Kim K. (2012). Functionalization of Graphene: Covalent and Non-Covalent Approaches, Derivatives and Applications. Chem. Rev..

[19] Kuila T., Bose S., Mishra A., Khanra P., Kim N., Lee J. (2012). Chemical functionalization of graphene and its applications. Prog. Mater. Sci..

[20] Bakandritsos A., Pykal M., Błoński P., Jakubec P., Chronopoulos D., Poláková K., Georgakilas V., Čépe K., Tomanec O., Ranc V. (2017). Cyanographene and Graphene Acid: Emerging Derivatives Enabling High-Yield and Selective Functionalization of Graphene. ACS Nano.

[21] Marcano D., Kosynkin D., Berlin J., Sinitskii A., Sun Z., Slesarev A., Alemany L., Lu W., Tour J. (2010). Improved Synthesis of Graphene Oxide. ACS Nano.

[22] Zbořil R., Karlický F., Bourlinos A., Steriotis T., Stubos A., Georgakilas V., Šafářová K., Jančík D., Trapalis C., Otyepka M. (2010). Graphene Fluoride: A Stable Stoichiometric Graphene Derivative and its Chemical Conversion to Graphene. Small.

[23] Chronopoulos D., Bakandritsos A., Pykal M., Zbořil R., Otyepka M. (2017). Chemistry, properties, and applications of fluorographene. Appl. Mater. Today.

[24] Li D., Müller M., Gilje S., Kaner R., Wallace G. (2008). Processable aqueous dispersions of graphene nanosheets. Nat. Nanotechnol..

[25] Su C., Loh K. (2012). Carbocatalysts: Graphene Oxide and Its Derivatives. Acc. Chem. Res..

[26] Eda G., Lin Y., Mattevi C., Yamaguchi H., Chen H., Chen I., Chen C., Chhowalla M. (2010). Blue Photoluminescence from Chemically Derived Graphene Oxide. Adv. Mater..

[27] Liu L., Wang L., Gao J., Zhao J., Gao X., Chen Z. (2012). Amorphous structural models for graphene oxides. Carbon.

[28] Shi H., Wang C., Sun Z., Zhou Y., Jin K., Redfern S., Yang G. (2014). Tuning the nonlinear optical absorption of reduced graphene oxide by chemical reduction. Opt. Express.

[29] Abid S.P., Islam S.S., Mishra P., Ahmad S. (2018). Reduced graphene oxide (rGO) based wideband optical sensor and the role of Temperature, Defect States and Quantum Efficiency. Sci. Rep..

[30] Nair R., Ren W., Jalil R., Riaz I., Kravets V., Britnell L., Blake P., Schedin F., Mayorov A., Yuan S. (2010). Fluorographene: A Two-Dimensional Counterpart of Teflon. Small.

[31] Zaoralová D., Hrubý V., Šedajová V., Mach R., Kupka V., Ugolotti J., Bakandritsos A., Medved’ M., Otyepka M. (2020). Tunable Synthesis of Nitrogen Doped Graphene from Fluorographene under Mild Conditions. ACS Sustain. Chem. Eng..

[32] Papadakis I., Bouza Z., Couris S., Bourlinos A., Mouselimis V., Kouloumpis A., Gournis D., Bakandritsos A., Ugolotti J., Zboril R. (2017). Hydrogenated Fluorographene: A 2D Counterpart of Graphane with Enhanced Nonlinear Optical Properties. J. Phys. Chem. C.

[33] Fedorov E.V., Grayfer E.D., Makotchenko V., Semenovich N.A., Shin H., Choi J. (2012). Highly Exfoliated Graphite Fluoride as a Precursor for Graphene Fluoride Dispersions and Films. Croat. Chem. Acta.

[34] Bourlinos A., Georgakilas V., Zboril R., Steriotis T., Stubos A. (2009). Liquid-Phase Exfoliation of Graphite towards Solubilized Graphenes. Small.

[35] Stavrou M., Dalamaras I., Karampitsos N., Couris S. (2020). Determination of the Nonlinear Optical Properties of Single- and Few-Layered Graphene Dispersions under Femtosecond Laser Excitation: Electronic and Thermal Origin Contributions. J. Phys. Chem. C.

[36] Papadakis I., Bakandritsos A., Swain A., Szabo T., Couris S. (2020). Effects of Size and Oxidation on the Nonlinear Optical Response and Optical Limiting of Graphene Oxide Sheets. J. Phys. Chem. C.

[37] Sheik-Bahae M., Said A.A., Wei T.H., Hagan D.J., Van Stryland E.W. (1990). Sensitive Measurement of Optical Nonlinearities Using a Single Beam. IEEE J. Quant. Electr..

[38] Dimiev A.M., Siegfied E. (2021). Graphene Oxide: Fundamentals and Applications.

[39] Trusovas R., Račiukaitis G., Niaura G., Barkauskas J., Valušis G., Pauliukaite R. (2015). Recent Advances in Laser Utilization in the Chemical Modification of Graphene Oxide and Its Applications. Adv. Opt. Mater..

[40] Trusovas R., Ratautas K., Račiukaitis G., Barkauskas J., Stankevičienė I., Niaura G., Mažeikienė R. (2013). Reduction of graphite oxide to graphene with laser irradiation. Carbon.

[41] Wu T., Liu S., Li H., Wang L., Sun X. (2011). Production of Reduced Graphene Oxide by UV Irradiation. J. Nanosci. Nanotechnol..

[42] Potsi G., Bourlinos A.B., Mouselimis V., Polakova K., Chalmpes N., Gournis D., Kalytchuk S., Tomanec O., Błonski P., Medved’ M. (2019). Intrinsic Photo-Luminescence of Amine-Functionalized Graphene Derivatives for Bioimaging Applications. Appl. Mater. Today.

[43] Guérin K., Pinheiro J., Dubois M., Fawal Z., Masin F., Yazami R., Hamwi A. (2004). Synthesis and Characterization of Highly Fluorinated Graphite Containing sp^2^ and sp^3^ Carbon. Chem. Mater..

[44] Ren M., Wang X., Dong C., Li B., Liu Y., Chen T., Wu P., Cheng Z., Liu X. (2015). Reduction and transformation of fluorinated graphene induced by ultraviolet irradiation. Phys. Chem. Chem. Phys..

[45] Pumera M., Sofer Z. (2017). Towards stoichiometric analogues of graphene: Graphane, fluorographene, graphol, graphene acid and others. Chem. Soc. Rev..

[46] Papadakis I., Bouza Z., Couris S., Mouselimis V., Bourlinos A. (2018). Dramatic Enhancement of the Nonlinear Optical Response of Hydrogenated Fluorographene: The Effect of Midgap States. J. Phys. Chem. C.

[47] Liaros N., Tucek J., Dimos K., Bakandritsos A., Andrikopoulos K., Gournis D., Zboril R., Couris S. (2016). The effect of the degree of oxidation on broadband nonlinear absorption and ferromagnetic ordering in graphene oxide. Nanoscale.

